# Carbon dot/polylactic acid nanofibrous membranes for solar-mediated oil absorption/separation: Performance, environmental sustainability, ecotoxicity and reusability

**DOI:** 10.1016/j.heliyon.2024.e25417

**Published:** 2024-02-16

**Authors:** Monica Torsello, Shani Ben-Zichri, Lucia Pesenti, Sisira M. Kunnath, Chiara Samorì, Andrea Pasteris, Greta Bacchelli, Noa Prishkolnik, Uri Ben-Nun, Serena Righi, Maria Letizia Focarete, Sofiya Kolusheva, Raz Jelinek, Chiara Gualandi, Paola Galletti

**Affiliations:** a Department of Chemistry “Giacomo Ciamician”, University of Bologna, Via Selmi, 2, 40126, Bologna, Italy; b Department of Chemistry, Ben Gurion University of the Negev, Beer Sheva, 84105, Israel; c Department of Biological, Geological and Environmental Sciences, University of Bologna, Via Sant’Alberto 163, 48123, Ravenna, Italy; d Interdepartmental Centre for Research in Environmental Sciences (CIRSA), University of Bologna, Via S. Alberto, 163, 48123, Ravenna, Italy; e INSTM UdR of Bologna, University of Bologna, Via Selmi, 2, 40126, Bologna, Italy; f Health Sciences and Technologies – Interdepartmental Center for Industrial Research (HST-ICIR), Alma Mater Studiorum - Università di Bologna, 40064, Ozzano dell’Emilia, Bologna, Italy; g Interdepartmental Center for Industrial Research on Advanced Applications in Mechanical Engineering and Materials Technology, CIRI-MAM, University of Bologna, Viale Risorgimento, 2, 40136, Bologna, Italy; h Department of Physics and Astronomy “Augusto Righi”, University of Bologna, Viale Carlo Berti Pichat, 6/2, 40126, Bologna, Italy

**Keywords:** Electrospinning, Polylactic acid, Carbon dots, Oil-water separation, Solar energy

## Abstract

Carbon dots (CDs) are promising photothermal nanoparticles that can be utilized in environmental treatments. They exhibit favorable physicochemical properties, including low toxicity, physical and chemical stability, photo-dependant reversible behaviour, and environmentally friendly synthesis using benign building blocks. Here, we synthesized innovative CDs/polylactic acid (PLA) electrospun composite membranes for evaluating the removal of hydrophobic compounds like long-chain hydrocarbons or oils in biphasic mixtures with water. The ultimate goal was to develop innovative and sustainable solar-heated oil absorbents. Specifically, we fabricated PLA membranes with varying CD contents, characterized their morphology, thermal, and mechanical properties, and assessed the environmental impact of membrane production according to ISO 14040 and 14044 standards in a preliminary “cradle-to-gate” life cycle assessment study. Solar radiation experiments demonstrated that the CDs/PLA composites exhibited greater uptake of hydrophobic compounds compared to pure PLA membranes, ascribable to the CDs-induced photothermal effect. The adsorption and regeneration capacity of the new CDs/PLA membrane was demonstrated through multiple uptake/release cycles. Ecotoxicity analyses confirmed the safety profile of the new adsorbent system towards freshwater microalgae, further emphasizing its potential as an environmentally friendly solution for the removal of hydrophobic compounds in water treatment processes.

## Highlights

• Polylactic acid electrospun membranes containing carbon dots are prepared.
• Photothermal properties depends on carbon dots content.
• Membrane's hydrophobic compound absorption is enhanced by photothermal effect.
• The environmental sustainability of the system is affected by carbon dots content.

## Graphical abstract

**Figure:**
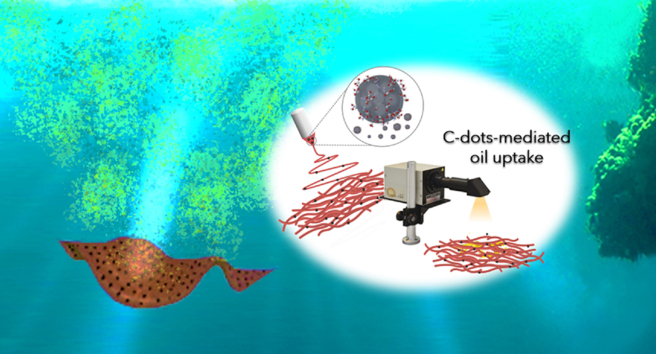

## 1 Introduction

Accidental oil spills and hydrophobic contaminants are a major cause of environmental pollution and pose a serious threat to ecosystems. Rapid and efficient removal of these pollutants is crucial to mitigate the potential long-term consequences and prevent further harm to the environment [[1], [2], [3]]. Innovative technologies and techniques have been developed to expedite the cleanup process, including membrane processes [4]. Ideal adsorbents should have high hydrophobicity and oleophilicity, high adsorption capacity and efficiency, high regeneration ability and low environmental impact.

Electrospun nanofibrous materials, known for their high surface area, exceptional porosity, outstanding mechanical properties, and ease of functionalization, are extensively studied for water remediation [5] and oil/water separation [[6], [7], [8]]. An advantage of employing electrospun membranes, as opposed to other materials such as aerogel, lies in the ease with which membrane porosity and fiber roughness can be controlled. These factors play a pivotal role in influencing the oil absorption capacity, distinguishing electrospun membranes as a favorable choice in such applications. Wu et al. [9] demonstrated this using electrospun polystyrene (PS) membranes with varied fiber diameters and porous fiber surfaces, highlighting the correlation between higher specific surface area and enhanced oil sorption capacity. Zhang et al. [10] achieved similar results with electrospun polylactic acid (PLA) incorporating γ-Fe_2_O_3_ nanoparticles, resulting in a dual-scale porous structure that significantly improved absorption capacity.

A further interesting aspect relies on the ease of functionalization of electrospun membranes which introduces new properties and enhances their adaptability to various oil separation applications. This can be achieved by incorporating additives during electrospinning or applying secondary modifications like coatings or plasma treatments. Wu et al. [11] successfully improved hydrophobicity and oil absorption capacity by incorporating fluorinated carbon nanotubes into PS fibers. Similar effects were observed with polyacrylonitrile fibers loaded with pitch [12]. Magnetic nanoparticles were introduced to polycaprolactone electrospun fibers to allow their fast and easy recovery from water surfaces using a magnetic field [13]. Surface modifications for superhydrophobicity were also explored [14,15].

Solar-heated oil absorbents, gaining recent interest for oil-water separation, leverage solar radiation to reduce oil viscosity and enhance thermal absorption. Various photothermal materials have been explored for this purpose, such as polyurethane-based photothermal sponges [16], composites containing carbon nanotubes [6], and other materials like polydiacetylene (PDA) [17] and carbon dots (CDs) [18]. CDs, carbonaceous nanoparticles synthesized through hydrothermal methods using various carbon-containing building blocks, display unique physical characteristics, including excitation-dependent luminescence, molecular imaging capabilities, diverse surface chemistries, and biocompatibility [19]. CDs serve as efficient photothermal agents for oil spill cleaning and solar-enabled water remediation. They can be incorporated into varying platforms, such as hydrogels, having tunable physico-chemical properties proven as great scaffolds for water harvesting and purification [20,21]. They can be integrated into many composites and efficiently reach the temperature at which oils become less viscous. Additionally, they also exhibit good water uptake, sunshine absorbance, and heat dissipation. For instance, the addition of CDs into commercial porous sponges may serve as novel solar-heating sorbent systems intended for crude oil spill remediation [22]. Recent studies show CDs incorporation into electrospun fibres for luminescent materials [23,24], likewise electrospun fibers combined with CDs can pave new routes in biomedical fields such as monitoring events or reporting potential diagnostics in biological systems. Last, CDs are also desirable for biomedical applications, such as scaffolds for bone tissue engineering as part of nanofibers systems and as drug delivery systems [[25], [26], [27], [28]].

Despite CDs are considered environmentally friendly, few researchers have explored the environmental impact of their production. Embedding sustainability assessment into product design is crucial, especially when proposing novel applications like incorporating CDs into electrospun membranes for oil-water separation [29].

In this context, Life Cycle Assessment (LCA) is the preferred tool for evaluating the environmental impact of green chemical processes and synthesis designs [30]. Existing literature on CDs production assesses environmental impacts using LCA methodology, typically employing a cradle-to-gate approach with 1 kg of CDs as the functional unit. Only one study attempts a cradle-to-grave assessment [31]. These studies discuss comparative LCAs focused on different synthesis routes [[32], [33], [34]], carbon precursors [35] or both [36,37]. All studies rely on lab data for the inventory.

In this work, we used electrospinning to produce membranes for oil-water separation with high surface area and high porosity. Leveraging the benefits of biobased poly-l-lactic acid (PLA), we imparted biodegradability to the membranes, obtaining good affinity with oil due to the inherent hydrophobic nature of the polymer. A key innovative aspect of our approach involved the incorporation of CDs through blend electrospinning, a simple and industrially exploitable production method. This strategy harnessed the photothermal effect, improving the oil uptake capacity of the membranes by employing clean and renewable energy. Different concentrations of CDs were used, while the building blocks used as precursors were kept constant. The resulting membranes underwent comprehensive characterization, evaluating their performance across different types of oils and in oil/water mixtures. The reusability was assessed over several cycles. To investigate the environmental impact, ecotoxicity tests were carried out through algal growth inhibition, verifying the ecotoxicity profile of the proposed composites. Furthermore, LCA was applied to assess the environmental profile of the CDs synthesis route. Subsequently, a comparative analysis was conducted to assess the environmental performance of producing PLA membranes loaded with CDs. The integration of these innovative elements not only enhances the performance of the membranes but also underscores the commitment to sustainable and environmentally conscious material design.

## 2 Experimental part

### 2.1 Materials

PLA (Lacea® H-100, M_w_ = 8.4 × 10^4^ g mol^−1^, PDI = 1.7) was provided by Mitsui Chemicals, Inc.). Dichloromethane (DCM, ACS reagent, ≥99.9 %), N,N-dimethylformamide (DMF, ACS reagent, ≥99.8 %), dimethylsulfoxide (DMSO, ACS reagent, ≥99.8 %), octadecylamine (ODA), urea, citric acid, castor oil, silicon oil AP 1000 and hexadecane were purchased from Sigma-Aldrich and used without further purification.

### 2.2 Carbon dots synthesis

CDs were prepared through the hydrothermal method as previously described [38]. Briefly, 1.0 g of citric acid, 1.0 g of ODA and 1.0 g of urea were dissolved in 10 mL DMSO solvent. The mixture was heated in a poly(tetrafluoroethylene) autoclave at 160 °C for 4 h. The obtained solution of CDs was centrifuged at 10000 rpm for 10 min and dialyzed against double distilled water (DDW) several times for purification. Finally, the sample was lyophilized obtaining dry CDs.

### 2.3 Membrane preparation by electrospinning

Nanofibrous mats were fabricated using a home-made electrospinning apparatus composed by an SL 50 P10/CE/230 high voltage power supplier (Spellman, New York, USA), a KDS-200 syringe pump (KD Scientific Inc., Massachusetts, USA), a glass syringe containing the polymer solution, a stainless-steel blunt-ended needle (inner diameter 0.5 mm, Hamilton, Bonaduz, Switzerland) connected with the power supply electrode and a grounded flat metal collector (8 × 8 cm^2^). Plain PLA membranes were produced from a 15 % (w/v) solution of PLA dissolved in a mixture of DCM:DMF = 70:30 (v/v). Proper amounts of CDs were added to the polymer solution to prepare CDs-doped membranes. Samples were labelled PLA-xCDs, where x indicates the weight content of CDs in the fibers (x = 5, 20 and 40). In a typical preparation, 0.75 g of PLA and 3.95 mg of CDs were mixed with 3.5 mL of DCM and 1.5 mL of DMF to prepare PLA-5CDs. The PLA solution and the CD dispersions were ejected at a flow rate of 1 mL h^−1^, the needle was placed at a distance of 20 cm from the collector and the applied voltage was set at 20–22 kV. Electrospinning was performed at room temperature (RT) and relative humidity of 30–40 %. The mean thickness of the plain PLA mat was 300 μm and that of the CDs-doped membranes was 150 μm.

### 2.4 Assessment of production sustainability

The LCA studies of the environmental performances of CDs and electrospun membrane productions were carried out according to ISO 14040 and 14044 standards [39,40]. The functional units (FUs) considered were 1 kg of CDs in the case of LCA for the CDs production and 3.2 ml of starting solution volume required for an electrospinning session (including the 0.2 mL of solution lost) in the case of LCA for the production of the CDs-doped membranes. The system boundaries were from “cradle-to-gate” in both cases. Life Cycle Inventory (LCI) is based on: laboratory-scale primary data, LCA datasets from GaBi Professional Database [41] and Ecoinvent Database version 3.8 [42], and literature data. In particular, ODA is absent in LCA databases and its inventory was created by following the chemical reaction between fatty alcohols and ammonia taken from the literature [43]; therefore inventory of ODA did not consider the energy consumption needed to carry out the reaction). The complete inventory data applied in the assessment are reported Tables S1 and S2. “INGEO Polylactide (PLA) biopolymer production” GaBi dataset (PLA made from corn grain) was selected as the best proxy of poly-l-lactic acid. CDs LCI was organized into upstream processes (precursor and solvent productions), core system processes (thermal decomposition, centrifugation, dialysis) and downstream processes (waste disposal). LCAs were performed using GaBi 10.0 modelling software. The impact assessment was performed using the ILCD V1.09 method that considers 16 impact categories: Acidification (Ac), Climate change (excluded CCex and included CCin biogenic carbon), Ecotoxicity freshwater (EcoF), Eutrophication freshwater (EuF), Eutrophication marine (EuM), Eutrophication terrestrial (EuT), Human toxicity (cancer and non-cancer, HTc and HTnc), Ionizing radiation (Ir), Land use (Lu), Ozone depletion (OD), Particulate matter (PM), Photochemical ozone formation (POF), Resource depletion water (WD), Resource depletion, mineral, fossils and renewables (RD).

### 2.5 Characterization methods

Emission fluorescence was carried out on a Fluorolog spectrofluorimeter (HORIBA, Japan). For spectra examination, the CDs were dispersed in chloroform at a concentration of 100 μg mL^−1^. Excitation wavelengths were in the range of 330–450 nm.

Atomic force microscopy (AFM) was carried out in tapping mode using an AC160 TS probe (CypherES, Oxford Instrument). CDs for AFM were prepared by drop-casting a dilute dispersion of CDs in chloroform (1 μg mL^−1^) on a silicon wafer and drying at room temperature.

UV–vis absorption spectroscopy was carried out using a Thermo Scientific Evolution 220 spectrophotometer on CD dispersions in chloroform (50 μg mL^−1^).

Fourier transform infrared spectroscopy (FTIR) of dried CDs and electrospun membranes was carried out through attenuated total reflectance (ATR)-FTIR using a Thermo Scientific, Nicolet 6700 spectrometer (USA). The IR spectra were recorded with 36 scans in the range of 4000-650 cm^−1^, with a resolution of 4 cm^−1^.

Scanning Electron Microscopy (SEM) was carried out using a Leica Cambridge Stereoscan 360 microscope at an accelerating voltage of 20 kV. Samples were sputter-coated with gold before examination and the distribution of fibre diameters was determined by measuring approximately 200 fibres using image analysis software (ImageJ). Results were presented as the average diameter ± standard deviation. The one-way ANOVA was used to test the statistical significance of the difference between the mean values (p < 0.01).

Thermogravimetric analysis (TGA) was carried out using a TA Instruments TGA Q500, from RT to 700 °C, using a heating rate of 10 °C min^−1^, in nitrogen atmosphere.

Differential Scanning Calorimetry (DSC) was performed using a TA Instruments Q100 DSC apparatus equipped with a Refrigerated Cooling System (RCS90). Samples were subjected to a first heating scan in the temperature range -90 - 240 °C at a rate of 20 °C min^−1^, followed by a controlled cooling at 10 °C min^−1^ to −90 °C and a second heating scan at 20 °C min^−1^ to 240 °C.

Static contact angle measurements were performed by using a Biolin Attension instrument under ambient conditions by recording the side profiles of deionized water drops for image analysis. The shape of the drop was recorded in a time range of 0–10 s, by collecting an image every 300 ms. Five drops were observed for each film and data were provided as average diameter ± standard deviation. WCAs were measured on electrospun membranes as well as on spin-coated films. The latter were obtained with a Laurell WS-650-23 Spin Coater (Laurell Technologies Corporation) by dissolving the electrospun membranes in chloroform and spin-coating the resulting solutions on a glass substrate.

Stress-strain measurements were performed with an Instron 4465 Testing Machine equipped with a 100 N load cell on rectangular specimens cut from electrospun mats. Samples were 5 mm wide, the gauge length was 20 mm and the cross-head speed was 5 mm min^−1^. Thickness was measured for each specimen by using a microcaliper. Load-displacement curves were obtained and converted to stress-strain curves. Five replicate specimens were run for each sample and mechanical data, Young's modulus (E), stress at break (σ_b_) and strain at break (ε_b_), were provided as the average value ± standard deviation.

### 2.6 Determination of CDs final concentration in membranes

Electrospun membranes were dissolved in DCM:DMF = 70:30 (v/v) solvent mixture at a concentration of 100 μg mL^−1^ and their fluorescence emission spectra were recorded (λ_ex_ = 420 nm, λ_em_ = 450–650 nm). Electrospun membranes were also dissolved in DCM:DMF = 70:30 (v/v) solvent mixture at a concentration of 330 μg mL^−1^ and their absorption emission spectra were recorded. A calibration curve at 450 nm for CDs was obtained by preparing a stock dispersion of CDs 330 μg mL^−1^ and by diluting the stock solution with DCM:DMF = 70:30 (v/v) to gain 9 dispersions of calibration standards (concentration range 1–100 μg mL^−1^). The CDs encapsulation efficiency (EE%) was calculated by applying Equation (1):

[1] $$EE\%=\frac{m_{a}}{m_{t}}\times 100$$

Where m_*a*_ corresponds to the actual amount of CDs in the membranes determined using the calibration curve, and m_*t*_ is the theoretical amount of C-dots according to the nominal feed. EE% was measured in triplicate for each CDs-doped membrane and values were provided as average ± standard deviation.

### 2.7 Membrane irradiation

Electrospun specimens (5 × 5 cm^2^) were irradiated by a solar simulator (Sciencetech, AX-LA125, ASTM Class-AAA) operating at a wavelength range of 350–1800 nm at 1 kW m^−2^ intensity. Experiments were carried out after dipping the membrane in castor oil, silicone oil or hexadecane. Samples were irradiated for 30 min by measuring the membrane temperature at intervals of 5 min using an IR camera (FLIR i7). Control experiments were performed by irradiating dry electrospun samples.

### 2.8 Oil absorption capacity

Oil absorption was tested for all the prepared membranes by using castor oil, silicone oil and hexadecane oil. Specimens of electrospun membranes (2 × 2 cm^2^) were placed in contact with 5 mL of pure oil for 30 min while irradiation was applied over the membrane by using the solar simulator. Control experiments were also carried out without applying radiation to the system. Similar experiments were also performed by testing hexadecane:H_2_O mixtures (75:25, 50:50 and 25:75 w/w). The oil absorption capacity (Q) of each membrane was determined as a weight increment, by measuring the weight of the membrane before and after oil/oil-water mixture contact and calculated according to Equation (2):

[2] $$Q=\frac{m_{f}-m_{i}}{m_{i}}$$

where m_*f*_ is the membrane weight after the experiment and m_*i*_ is the initial membrane weight. Q was determined for each membrane on five replicas and values were provided as average ± standard deviation. Electrospun membranes were regenerated by applying a cleaning procedure that encompasses washing with cyclohexane overnight to remove the absorbed oil and tested again in triplicate against oil absorption, as described above. Five cycles of use and regeneration were applied.

### 2.9 Algal growth inhibition assay with *Raphidocelis subcapitata*

As a preliminary assessment of the ecotoxicity of CDs/PLA composites, a membrane leachate was tested in a growth inhibition assay with unicellular green algae. The leaching test was intended to simulate the use of the membranes in aquatic environments. Unicellular algae were chosen because an ecologically relevant endpoint (population growth) could be measured, using small volumes of leachate and, consequently, small quantities of membranes. A leachate of the electrospun membranes containing 5 wt% of CDs (PLA-5CDs, cut in pieces of 0.5 × 0.5 cm^2^ size) was prepared in deionized water, using a sample-water ratio of 80 g L^−1^, with a leachate volume of 25 mL. The sample was shaken in a rotating incubator (125 rpm) at room temperature for 14 days in the dark [44]. After that time, the leaching solution containing free CDs was separated by pipetting after decanting the electrospun membranes and then used for the algal growth inhibition test. An aliquot of the leachate was withdrawn, freeze-dried, resuspended in CH_2_Cl_2_ and then analyzed by UV-spectroscopy using a UV/VIS Jasco 7800 spectrophotometer, against a blank with CH_2_Cl_2_. The electrospun membrane after leaching was dried under vacuum for 48 h and then analyzed in terms of elemental analysis using the flash combustion technique (Thermo Scientific, Flash2000, Organic Elemental Analyzer) and TGA. The algal growth inhibition test was conducted following the OECD 201 guideline [45], adapted to the use of 24-well plates as test vessels. The test organism was the freshwater unicellular green alga *Raphidocelis subcapitata*, strain SAG 61.81 (formerly named *Pseudokirchnerialla subcapitata*), originally purchased from EPSAG Göttingen University and cultivated in the laboratory. In each well, 1.5 mL of leachate, diluted with distilled water were mixed with 0.5 mL of concentrated culture medium and inoculated with an aliquot of algae from an exponentially growing culture. By diluting the leachate, eight concentrations arranged in a geometric progression from 0.5 % to 75 % were prepared, each in three replicates. Six replicates of the control treatment were also prepared (1.5 mL of distilled water + 0.5 mL of concentrated culture medium and algae). The plates were incubated for 72 h at 23 ± 2 °C on an orbital shaker at 100 rpm under continuous “cool white” fluorescent light (intensity of 6000 lx). The algal density (cell mL^−1^) in each well, at the beginning and at the end of the incubation, was determined by counting the number of cells in a sample of known volume, under a microscope at 400x, with a Burker hemocytometer. The average specific growth rate μ (d^−1^) of the algal population in each well was calculated, assuming exponential growth, according to Equation (3):

[3] $$\mu =\frac{\ln\;N_{f}-\ln\;N_{i}}{t}$$

where: N_*i*_ is the initial algal density, N_*f*_ is the final algal density at test termination, and t is the test duration. Differences in specific growth rates between the control and each concentration were evaluated using Dunnett's test and considered statistically significant if P < 0.05. The toxicity of the leachate was expressed as EC10 and EC50, the concentrations causing respectively a 10 % and a 50 % reduction in the algal growth rate in comparison to the control. EC10 and EC50 were estimated by fitting a three-parameter log-logistic function to the experimental concentration-effect data, using the drc package in R [46].

## 3 Results and discussion

### 3.1 Membrane characterization

**Fig. 1:**
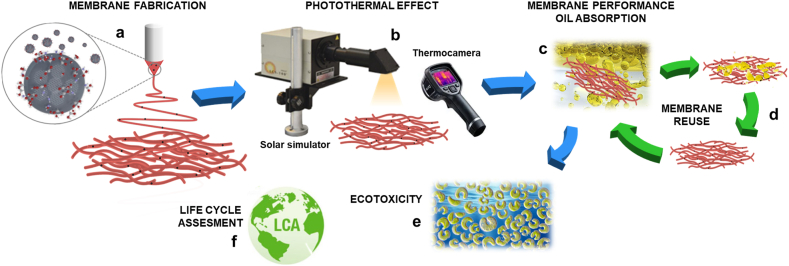
Sketch outlining the system under investigation: (a) Electrospun membranes, doped with varying amounts of CDs, were produced using blend electrospinning. (b) The membranes were tested under solar irradiation to assess the photothermal effect. (c) The irradiated membranes were tested against oil absorption capacity. (d) Electrospun membranes were regenerated and reused for five cycles. (e) Membrane ecotoxicity was tested against unicellular green alga. (f) Life Cycle Assessment was carried out on both CDs and electrospun membranes.

Electrospun membranes with different amounts of CDs were obtained through blend electrospinning, as illustrated in Fig. 1 and Fig. S1. This process involves dispersing the proper concentration of CDs in the polymeric solution. SEM images of electrospun PLA, PLA-5CDs, PLA-20CDs, and PLA-40CDs are shown in Fig. S2. After the optimization of the electrospinning process, we obtained smooth bead-free fibres with average diameters of 770 (±330) nm, 740 (±420) nm, 620 (±280) nm and 720 (±280) nm for PLA, PLA-5CDs, PLA-20CDs, PLA-40CDs membranes, respectively. The addition of CDs did not affect the fiber dimension (p > 0.05), with the exception of PLA-20CDs which displays a statistically significant reduction in fiber diameter (p < 0.01). Similarly, the surface morphology of the fibres appears unchanged, suggesting a homogeneous distribution of the particles in the fibres, except for PLA-40CDs membranes, where the fibres appear rougher due to the high content of CDs in this sample. CDs, employed as additives in the fibres, exhibited significant light absorbance across the entire UV–Vis range (Fig. S3a), confirming their ability to promote high heat dissipation throughout the photothermal effect. Fig. S3b reports the emission spectra of CDs obtained at different excitation wavelengths, confirming the wavelength-dependent behaviour of the emission, typical of such materials [43]. AFM analysis shows that CDs have diameters of about 15 nm (Fig. S3c).

**Fig. 2:**
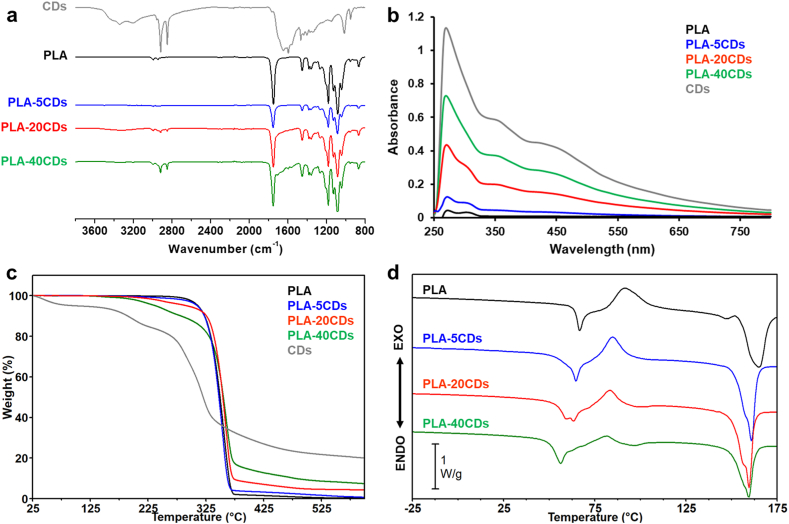
(a) ATR-FTIR spectra. (b) UV–Vis spectra (membranes solubilized in DCM:DMF = 70:30 v/v). (c) TGA curves of PLA (black), CDs (grey), PLA-5CDs (blue), PLA-20CDs (red), and PLA-40CDs (green). (d) DSC first heating scan of PLA (black), CDs (grey), PLA-5CDs (blue), PLA-20CDs (red), and PLA-40CDs (green).

The FTIR spectra of the various membranes are shown in Fig. 2a. In CDs, the peak at 3400 cm^−1^ is ascribable to O–H stretching, with narrow peaks overlapping the broad peak of –OH related to the N–H stretching. Peaks in the region 3000-2800 cm^−1^ are due to the symmetric and asymmetric stretching vibration of C–H bond in alkyl groups, and the broad peak located in the 1700-1600 cm^−1^ region is possibly due to aromatic C=C stretching and C=O stretching. These characteristic peaks reveal the existence of amino, hydroxyl, carboxyl and alkyl groups in CDs, consistent with previous works [18,20].

All PLA-based membranes share IR peaks at 1361 cm^−1^ and 1730 cm^−1^ ascribable, respectively, to the C–O stretching of PLA and to the C=O stretching found in both PLA and CDs (Fig. 2a, blue curve). In addition, all PLA-based membranes show a shoulder in the 1700-1600 cm^−1^ region, with intensities increasing with CDs content.

The effective loading of CDs in the fibres was quantitatively determined by UV–Vis absorbance spectra of a known amount of membrane dissolved in organic solvent (Fig. 2b, the calibration curve is reported in Fig. S4a). The rising absorbance intensity with the increase of the nominal concentration of CDs was accompanied by a concomitant increase in the emission intensity (Fig. S4b). The calculated CDs loadings were 1.2 (±0.2) wt %, 12.7 (±0.9) wt % and 34.6 (±0.6) wt % for PLA-5CDs, PLA-20CDs and PLA-40CDs, respectively. It is worth noting that the actual CDs concentration in the fibres was lower than the nominal one for all the tested samples, possibly justified by a certain tendency of the CDs to settle in the syringe during the electrospinning process, albeit this hypothesis was difficult to verify since all electrospun solutions loaded with CDs appeared black in the course of the process.

Thermogravimetric analysis was carried out to evaluate the effect of CDs on the thermal stability of PLA membranes (Fig. 2c). It can be observed that the weight of all membranes remained stable up to 200 °C, indicating the absence of low boiling substances, such as residual solvents from the electrospinning process, within the detectability limits of TGA. PLA starts to degrade above 250 °C, with a single weight loss that ends at around 500 °C, showing a maximum degradation temperature (T_max_) at 355 °C. CDs show a first weight loss at low temperatures, suggesting the presence of residual solvent in the lyophilized material. After that, the CDs degradation involves a two-step process and leads to a 20 % residual weight at 600 °C, in line with previous findings [47]. The incorporation of CDs in the membranes anticipates the onset of the degradation process without altering the maximum temperature of degradation. As the CDs content increases, there is a noticeable rise in residue at the end of the analysis. Based on the residual weight at 600 °C, i.e. 0.4 %, 0.5 %, 4.3 % and 7.4 % for PLA, PLA-5CDs, PLA-20CDs and PLA-40CDs, respectively, the actual content of CDs in the doped membranes was estimated to be 3.5 %, 21.5 % and 37.0 % respectively for PLA-5CDs, PLA-20CDs and PLA-40CDs. It is pointed out that fluorescence spectroscopy is a more reliable method for CDs quantification, being residual weight in TGA very small this introduces high uncertainty in the final result.

DSC analysis was performed to evaluate the effect of CDs doping on the thermal transitions of the fibres (Fig. 2d–Table 1). Electrospun membranes show an endothermal step-change in the baseline, ascribable to the glass transition temperature (T_g_), along with a cold crystallization exothermal peak, followed by the endothermal melting peak. In the first scans, T_g_ values are challenging to measure since T_g_ is accompanied by an enthalpic relaxation peak associated with physical ageing. The comparison of cold crystallization (ΔH_c_) and of melting (ΔH_m_) enthalpies highlighted that the pure PLA membrane after electrospinning is completely amorphous, since ΔH_c_ = ΔH_m_, in line with previously reported data [48,49]. Differently, in all CDs-doped membranes, ΔH_c_ is lower than ΔH_m_, suggesting the development of a certain fraction of crystal phase during the electrospinning process. In addition, as the amount of CDs increases, the difference between the enthalpy of cold crystallization and melting increases, suggesting a possible nucleating effect of the CDs by promoting the crystallization process of PLA [50,51]. This result was further confirmed by the analysis of melting enthalpy values of the samples calculated with respect to the content of PLA, ΔH_m_ (PLA) (Table 1), indicating that PLA crystallizes at a greater extent in the presence of CDs. After erasing the sample thermal history and applying a controlled cooling, sample T_g_ can be determined and, as the content of CDs increases, a slight anticipation in T_g_ is noticed. Specifically, the T_g_ decreases from 59 °C for pure PLA to 56 °C for PLA-5CDs and to 53 °C for PLA-20CDs, while T_g_ is not detectable in PLA-40CDs sample. The decrease of polymer T_g_ might indicate a plasticizing effect of the CDs.

**Table 1:** Calorimetric data of DSC heating scans of CDs and electrospun membranes.

	1st heating scan					
Sample	T_g_ [°C]	T_c_ [°C]	ΔH_c_ [J g^−1^]	T_m_ [°C]	ΔH_m_^a^ [J g^−1^]	ΔH_m_ (PLA)^b^ [J g^−1^]
PLA	n.d.^c^	92	38.4	165	38.9	38.9
PLA-5CDs	n.d.^c^	85	34.4	161	37.2	39.2
PLA-20CDs	n.d.^c^	83	24.5	160	37.2	46.5
PLA-40CDs	n.d.^c^	82	16.5	160	28.4	47.3
	Heating scan after controlled cooling					
Sample	T_g_ [°C]	T_c_ [°C]	ΔH_c_ [J g^−1^]	T_m_ [°C]	ΔH_m_^a^ [J g^−1^]	ΔH_m_ (PLA)^b^ [J g^−1^]
PLA	59	121	34.8	159	38.3	38.3
PLA-5CDs	56	107	17.5	159	43.3	45.6
PLA-20CDs	53	104	10.0	156	44.8	56.0
PLA-40CDs	n.d.^c^	104	7.0	153	32.7	54.5

a) Melting enthalpy per gram of whole sample.

b) Melting enthalpy per gram of PLA, determined by applying the following equation: ${\Delta H}_{m}\left(\text{PLA}\right)=\frac{{\Delta H}_{m}}{w^{\text{PLA}}}$, where ${\Delta H}_{m}$ is the melting enthalpy of the sample and $w^{\text{PLA}}$ is the nominal weight fraction of PLA in the membrane.

c) n.d.: not detectable.

**Fig. 3:**
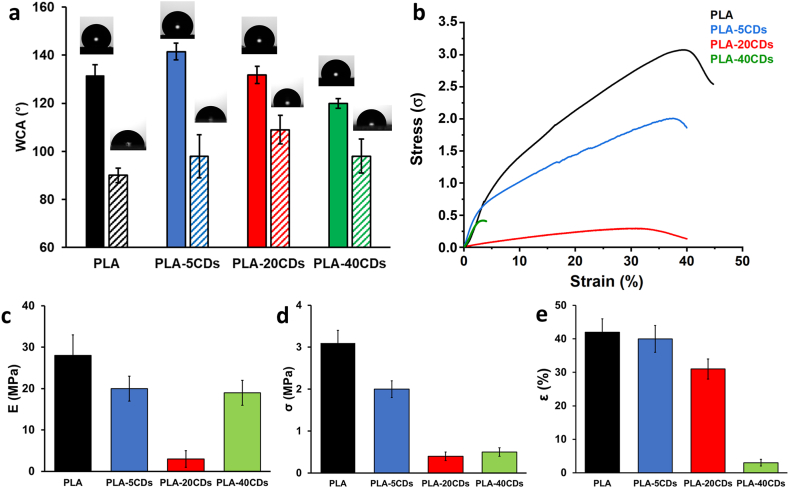
Wettability and mechanical properties of PLA (black), PLA-5CDs (blue), PLA-20CDs (red), PLA-40CDs (green). a) WCA of electrospun membranes (filled bars) and spin-coated films (half-filled bars); b) representative stress-strain curves of electrospun membranes; c) elastic modulus (E); d) stress at break (σ_b_); e) strain at break (ε_b_).

Water contact angles of electrospun membranes were measured to investigate the influence of CDs on the hydrophobicity of the samples. Fig. 3a shows that the addition of 5 wt% of CDs led to a slight increase in WCA, although this effect was not observed at higher CD contents. It is essential to emphasize that the wettability of electrospun membranes is influenced not only by fiber composition but also by fiber roughness and mat porosity. These findings can be attributed to the micro-scale dimensions of the mat pores and are consistent with the predictions of the Cassie-Baxter theory, which relates the different water contact angles of the bulk and the electrospun mat surface to the presence of trapped air within the structure [52,53]. To eliminate the influence of membrane morphology and accurately evaluate the impact of CDs on sample hydrophobicity, we measured the WCA of corresponding films (Fig. 3a). As expected, the smooth films exhibited lower water contact angle values compared to their corresponding electrospun mats, as previously observed in other hydrophobic polymers [54,55]. Notably, an increase in film hydrophobicity was observed with the addition of CDs up to 20 wt%. A similar contribution of CDs to the hydrophobicity is expected in the fibers; however, the mere measurement of WCA on the electrospun membrane cannot provide this information as the morphology factor masks the effect of the material.

Fig. 3b–e and Table 2 report representative stress-strain curves and mechanical data of the investigated samples, respectively. A clear decrease in Young's modulus (E), stress at break (σ_b_) and deformation at break (ε_b_) is observed with the increase of CDs loading, up to CDs concentration of 20 wt%. These results are unexpected since, typically, the inclusion of carbonaceous nanomaterials in polylactic acid enhances the strength and the rigidity of the resulting composite, albeit this effect is achieved at loadings well below 5 wt% [56]. A reduction in E has been previously observed with other fillers, in line with our results, and is ascribed to a plasticization effect of the filler [57,58]. PLA-40CDs is out of this trend, displaying a Young's modulus similar to that of PLA-5CDs and a remarkably lower deformation at break, which is an indication of the high fragility of this sample, possibly ascribable to CDs aggregation along the fibres. The unexpected trend of mechanical properties is challenging to rationalize, especially considering that the mechanical properties of nonwoven electrospun structures depend not only on the individual fibre features but also on the arrangement of the fibres in the nonwoven matrices, such as fibre direction and curvature, fibre interconnections, fibre fusion at contact points and mat porosity [59,60].

**Table 2:** Young's modulus (E), stress at break (σ_b_), and deformation at break (ε_b_) of the electrospun samples were determined by stress-strain tests.

Sample	E [MPa]	σ_b_ [MPa]	ε_b_ [%]
PLA	28 ± 5	3.1 ± 0.3	42 ± 4
PLA-5CDs	20 ± 3	2.0 ± 0.2	40 ± 4
PLA-20CDs	3 ± 2	0.4 ± 0.1	31 ± 3
PLA-40CDs	19 ± 3	0.5 ± 0.1	3 ± 1

### 3.2 Life cycle assessment

The environmental impacts obtained through LCA concerning CDs and PLA-based membrane preparation are reported as “environmental profile”, which comprises scores reflecting the potential effects on different environmental and health issues (with higher scores indicating a greater environmental impact). The environmental profile of CDs synthesis shows that the core-system processes exert the most significant impact across all impact categories, except for ozone depletion (Table 3). In particular, the power consumption associated with the dialysis process almost always stands out as the predominant contributor to the environmental impact, accounting for more than 80% of the total impact in most cases. This implies that a substantial reduction in power consumption during dialysis could result in one order of magnitude decrease in the impact score of many categories. The main contribution to ozone depletion is from upstream processes, in particular the ODA production, constituting approximately 70% of the total impact. Downstream processes always make minimal contributions to the scores of the environmental profile.

**Table 3:** Environmental profile of CDs synthesis (FU: 1 kg; system boundaries: cradle-to-gate). The meaning of acronyms can be found in section 2.4.

Impact category	Units	Total	Upstream processes	Core system processes	Downstream processes
AC	mol H^+^ eq	4.06E+01	3.30E-01	4.02E+01	3.40E-02
CCex	kg CO_2_ eq	1.92E+04	9.36E+01	1.91E+04	1.98E+01
EcoF	CTUe	1.39E+03	4.53E+02	8.82E+02	5.55E+01
EuF	kg P eq	6.80E-02	7.80E-03	5.33E-02	6.88E-03
EuM	kg N eq	1.02E+01	4.89E-02	1.01E+01	5.54E-02
EuT	kg N eq	1.01E+02	6.52E-01	1.00E+02	1.37E-01
HTc	CFUh	2.35E-05	2.36E-06	1.85E-05	2.71E-06
HTnc	CFUh	1.02E-03	1.08E-05	7.71E-04	2.38E-04
Ir	kBq U235 eq	8.41E+03	4.99E+00	8.41E+03	8.59E-01
Lu	Pt	9.49E+03	4.41E+01	9.42E+03	3.48E+01
OD	kg CFC11 eq	1.57E-05	1.56E-05	1.26E-09	2.02E-13
PM	kg PM2.5 eq	1.99E+00	8.34E-02	1.90E+00	1.26E-03
POF	kg NMVOC eq	2.60E+01	1.90E-01	2.58E+01	3.35E-02
WD	m^3^ eq	1.33E+03	1.12E+00	1.33E+03	4.43E-01
RD	kg Sb eq	6.59E-02	1.10E-03	6.49E-02	−5.23E-06

The environmental impacts of electrospun membranes of PLA, PLA-5CDs, PLA-20CDs and PLA-40CDs were calculated and reported in Table 4. As it is possible to observe, the addition of CDs increases the score: the greater the CDs content, the higher the environmental impact. PLA-40CDs can show impacts that can be one order of magnitude higher than those observed for PLA and PLA-5CDs.

**Table 4:** Environmental profile of PLA-based membranes synthesis (FU: 3.2 ml of starting solution; system boundaries: cradle-to-gate). The meaning of acronyms can be found in section 2.4.

Impact category	Units	PLA	PLA-5CDs	PLA-20CDs	PLA-40CDs
AC	mol H^+^ eq	1.48E-03	1.52E-03	5.28E-03	1.29E-02
CCex	kg CO_2_ eq	6.93E-01	6.75E-01	2.37E+00	5.79E+00
EcoF	CTUe	1.02E-01	1.08E-01	2.30E-01	4.88E-01
EuF	kg P eq	3.02E-06	3.25E-06	9.67E-06	2.29E-05
EuM	kg N eq	3.77E-04	3.70E-04	1.27E-03	3.09E-03
EuT	kg N eq	3.66E-03	3.56E-03	1.24E-02	3.03E-02
HTc	CFUh	1.69E-09	1.71E-09	3.85E-09	8.18E-09
HTnc	CFUh	3.88E-08	3.67E-08	1.29E-07	3.18E-07
Ir	kBq U235 eq	2.89E-01	3.17E-01	1.15E+00	2.84E+00
Lu	Pt	3.37E-01	3.67E-01	1.30E+00	3.19E+00
OD	kg CFC11 eq	2.46E-07	2.62E-07	2.63E-07	2.67E-07
PM	kg PM2.5 eq	7.43E-05	7.63E-05	2.60E-04	6.33E-04
POF	kg NMVOC eq	1.24E-03	1.19E-03	3.50E-03	8.08E-03
WD	m^3^ eq	4.58E-02	4.37E-02	1.59E-01	3.91E-01
RD	kg Sb eq	2.32E-06	2.49 E−06	8.88 E−06	2.18 E−05

**Fig. 4:**
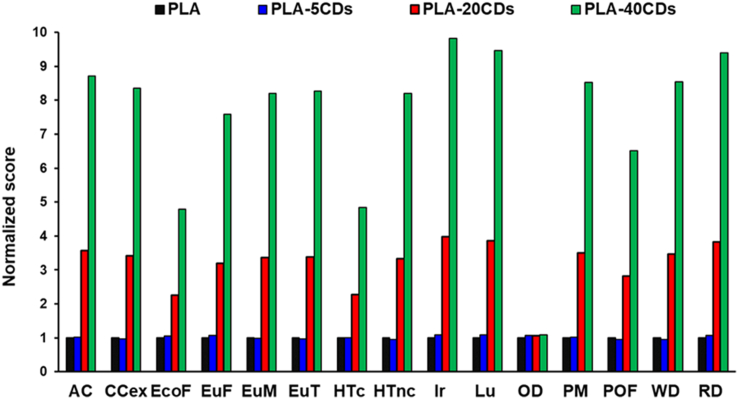
Environmental profiles of PLA-5CDs (blue), PLA-20CDs (red) and PLA-40CDs (green) normalized with respect to unloaded PLA membrane (black). The meaning of acronyms can be found in section 2.4.

Fig. 4 reports the environmental profiles of PLA, PLA-5CDs, PLA-20CDs and PLA-40CDs normalized with respect to the unloaded PLA membrane. This normalization allows for a comparison across the different impact categories and the different membranes. It is evident that the scores for PLA-5CDs are very similar to those of the unloaded PLA; on the contrary, the addition of higher quantities of CDs significantly increases the environmental impact. The environmental impact categories less affected by doping with CDs versus undoped PLA membrane include ozone depletion, human toxicity (cancer and no cancer) and ecotoxicity.

### 3.3 Oil uptake and oil/water separation

**Fig. 5:**
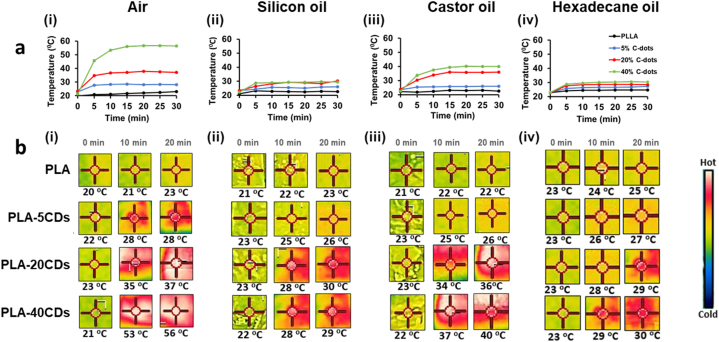
(a) Temperature behaviour of PLA (black), PLA-5CDs (blue), PLA-20CDs (red), PLA-40CDs (green) membranes as a function of irradiation time both in air and in presence of different oils. (b) Temperature maps of the tested membranes in the different conditions and irradiation time: i) dry conditions; ii) silicon oil; iii) castor oil; iv) hexadecane.

CDs doped membranes were exposed to 1 kW m^−2^ of solar simulator illumination and the temperatures of the various membranes were recorded at 5 min intervals over a total of 30 min min using an infrared (IR) thermocamera (Fig. 5). The irradiation of membranes in air (Fig. 5a (i) and 5b (i)), without the presence of oil, results in a dramatic increase in temperature of the CDs-doped membranes, which seems related to the nominal content of CDs, when compared to the undoped PLA membrane. This observation underscores the crucial role of CDs in promoting solar-induced heating through effective light absorption.

Solar simulation irradiation was carried out also in the presence of different oils: silicon oil (Fig. 5a (ii) and 5b (ii)) and castor oil (Fig. 5a (iii) and 5b (iii)), frequently used as model systems in oil cleanup tests [61,62], while hexadecane (Fig. 5a (iv) and 5b (iv)) can be used as a model to simulate a genuine situation involving hydrocarbon-based pollutants. The photothermal effect of CDs is clearly evident also when membranes are impregnated in the tested oils, albeit, with respect to dry conditions, the temperature increase is significantly lower due to heat dissipation. The differences in temperature reductions for the different types of oil (Fig. 5(ii)-(iv)) can be ascribed to the variance in oil densities and chemical properties, leading to different heat uptake by the oils absorbed within the membranes. In addition, it is evident that CDs contribute to effectively increasing the temperature of oil-impregnated membranes only up to 20 wt% loadings.

**Fig. 6:**
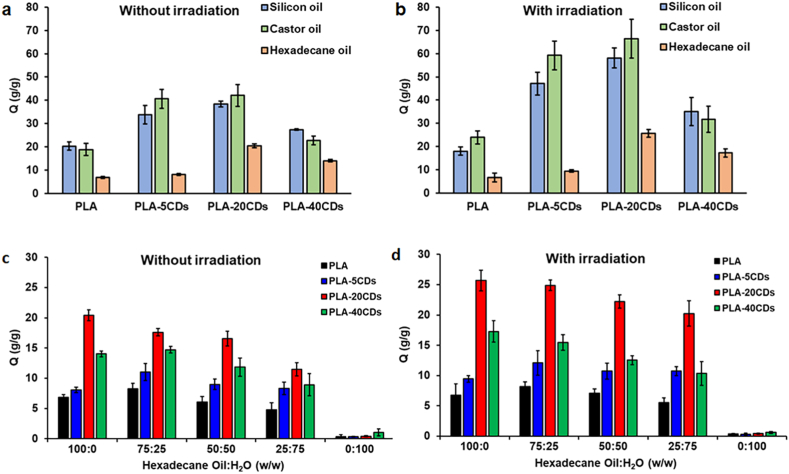
Oil absorption capacity (Q) of electrospun membranes against different types of oil at room temperature (a) and with irradiation (b). Hexadecane oil absorption capacity of electrospun membranes in oil/water mixtures without (c) and with irradiation (d).

Interestingly, the oil uptake data reported in Fig. 6a shows that the ability of CDs-doped membranes to absorb silicone oil (blue bars), castor oil (green bars), and hexadecane (orange bars) increases as a function of the nominal content of CDs up to 20 %w/w. It is worth noting that the mere presence of CDs, without solar irradiation, enhances the oil absorption capacity, likely owing to the highly hydrophobic nature of the CDs, which improves the membrane affinity with all the tested oils, in line with WCA data. For instance, undoped PLA absorbs 20 times its weight of silicon oil while the PLA-20CDs can absorb nearly 40 times its weight of silicon oil. A further increase in oil absorption capacity is observed for photoactivated membranes (Fig. 6b). This improvement can be reasonably ascribed to a reduction in oil viscosity promoted by the rising temperature that encourages oil uptake into the pores of the membranes. Notably, loading 40 % of CDs induces a dramatic decline in the oil capacity. This finding can be influenced both by the presence of many CDs aggregates, which results in lower hydrophobization compared to PLA-20CDs (consistent with WCA data), and by the high fragility of the PLA-40CDs membrane which makes a reliable determination of the oil uptake difficult.

Oil uptake tests were also performed on oil/water mixtures at different concentrations using hexadecane to simulate real conditions (Fig. 6c and d). These tests confirmed that PLA-20CDs displays the highest oil absorption capacity among the tested membranes, both in the absence of solar radiation (Fig. 6c) and under irradiation (Fig. 6d). The outcomes of membrane oil absorbance ability were in line with the data related to pure oil matrices, indicating that solar irradiation improves the oil uptake also in an oil/water mixture.

**Fig. 7:**
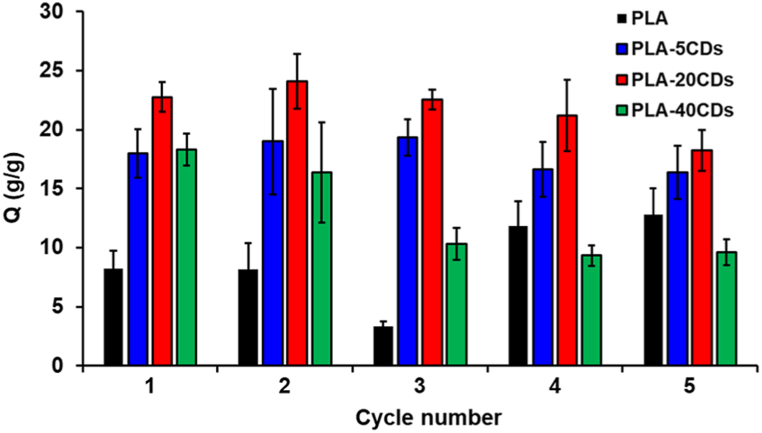
Hexadecane absorption capacity of the electrospun membranes over five cycles after membrane cleaning with cyclohexane.

In a further experiment, the possibility of regenerating and reusing the membranes was assessed in hexadecane by removing the absorbed oil after an oil absorption test and exposing the membranes again to hexadecane. The test was repeated 5 times and the results are reported in Fig. 7. All membranes maintained their ability to absorb hexadecane, and the oil absorption capacity remained constant also after five cycles of regeneration and reuse, the only exception being PLA-40CDs, whose fragility poses limitations to its use. This result demonstrates the potentiality of the proposed approach.

### 3.4 Ecotoxicity evaluation

With a view towards an application in aquatic environments, PLA-5CDs were subjected to a leaching test to ascertain their stability and CDs release in water. After 24 h, the water solution had a pale yellow colour which became more intense over the next 24 h (see ESI); at the end of the leaching test (14 d) the electrospun membrane was intact but with a lower C content than the membranes before the leaching (50.0 ± 0.1 % vs 52.6 ± 0.4 %), suggesting the release of the CDs. This hypothesis was confirmed by the dark colour of the water solution (Fig. S5b) and its UV absorbance spectrum (Figs. S5c and d). In parallel, the TGA analysis of the retrieved membrane shows a clear decrease in its thermal stability compared to the unexposed membrane (Fig. S5e). Since the thermal stability of polyesters depends on their molecular weight [63], this decrease in thermal stability may suggest the occurrence of polymer hydrolytic degradation during the 14 d experiment and confirms the capability of PLA to be degraded in an aqueous environment.

**Fig. 8:**
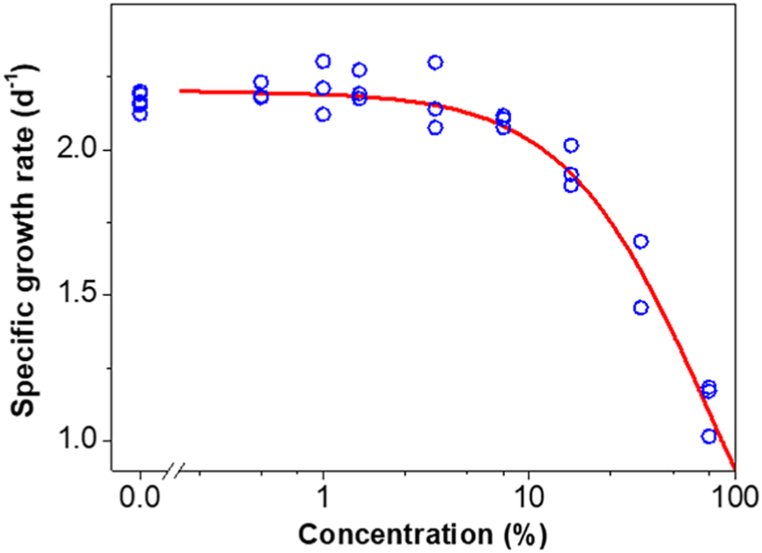
Effect of leachate of electrospun PLA-5CDs on the specific growth rate of the green microalga *Raphidocelis subcapitata*. The continuous line represents the log-logistic model fitted to the observed data.

The leachate was then tested in an algal growth inhibition test to verify its potential hazard towards photosynthetic biological targets. The effect of the leachate on the algal-specific growth rate was clearly concentration-dependent (Fig. 8), without apparent inhibition at the five lowest tested concentrations (0.5–7.5 %). The LOEC, i.e. the lowest concentration that caused a statistically significant reduction in the specific growth rate, compared to the control was 16 %. The estimated EC10 was 13.7 % (95% confidence interval: 9.9 %–16.3 %). The estimated EC50 was 75 % (67 %–84 %).

These results indicate that the release of biologically active substances in the aquatic environment from CDs- PLA membranes cannot be excluded. However, the estimated toxicity values indicated that inhibition of the specific growth rate was detectable only above 10 % of leachate. This corresponds to a membrane-water ratio of 8 g L^−1^. The actual ratio attainable in the aquatic environment will depend on the use and disposal of the membranes, but, in any case, the order of magnitude of g L^−1^ seems unrealistic [64]. The dark colour of the leachate alone, by decreasing the light penetration in the water column and, consequently, the photosynthesis, could explain the observed growth inhibition. Leachates obtained from polypropylene (PP), polystyrene (PS) and polyvinyl chloride (PVC), using the same ratio of 80 g L^−1^ and methods comparable to those used in the present study, demonstrated higher growth inhibition on *R. subcapitata*, with EC50s from 1.62 % (PVC) to 64 % (PP). Polyethylene terephthalate (PET), on the other hand, did not cause any growth inhibition [44]. Reported EC50s of carbon dots for growth inhibition of unicellular algae range from 5 to 250 mg L^−1^ [[65], [66], [67], [68]]. This variation is arguably due to the differences in chemical composition, size and structure of the tested CDs and the different algae species used in the assays. However, the available data do not allow the identification of any general pattern.

## 4 Conclusion

In this study, we developed new hydrophobic nanofiber membranes doped with CDs for oil spill remediation. The CDs incorporated into the membranes harness sunlight-induced heating to facilitate effective oil absorption. In particular, the enclosed CDs are the key chemical components responsible for solar-mediated oil uptake. Carbon nanoparticles convert absorbed solar energy into heat, leading to an increase in temperature that reduces oil viscosity, thereby facilitating oil diffusion into the nanofiber membranes. Compared to previously reported methods, the new solar-mediated oil clean-up procedure proposed in this work offers significant advantages. The nanofiber membranes and CDs composites were produced using sustainable starting materials. Moreover, the fabrication of CDs is based on a simple method suitable for mass production, albeit doping with CDs significantly increases the environmental impact of electrospun membranes proportionally to the weight of CDs. The application of the nanofiber membranes-CDs system for the removal of highly viscous oils, a challenging task in most existing technologies, is made possible by the exceptional photothermal characteristics of CDs. Overall, these new composites have the potential to be efficient tools for cleaning up oil spills due to their straightforward synthesis, superior thermal and mechanical stability and recycling capabilities.

## Data availability statement

The raw/processed data required to reproduce these findings cannot be shared at this time as the data also forms part of an ongoing study.

## CRediT authorship contribution statement

**Monica Torsello:** Writing – review & editing, Writing – original draft, Visualization, Investigation, Formal analysis. **Shani Ben-Zichri:** Writing – original draft, Visualization, Investigation, Formal analysis. **Lucia Pesenti:** Investigation, Formal analysis. **Sisira M. Kunnath:** Investigation. **Chiara Samorì:** Writing – review & editing, Writing – original draft, Methodology, Investigation, Conceptualization. **Andrea Pasteris:** Writing – original draft, Methodology, Investigation. **Greta Bacchelli:** Investigation, Formal analysis. **Noa Prishkolnik:** Investigation. **Uri Ben-Nun:** Investigation. **Serena Righi:** Writing – review & editing, Writing – original draft, Methodology, Conceptualization. **Maria Letizia Focarete:** Supervision, Conceptualization, Writing – review & editing. **Sofiya Kolusheva:** Supervision, Methodology, Conceptualization. **Raz Jelinek:** Funding acquisition, Conceptualization. **Chiara Gualandi:** Writing – review & editing, Writing – original draft, Visualization, Supervision, Methodology, Conceptualization. **Paola Galletti:** Methodology, Funding acquisition, Conceptualization, Writing - review & editing.

## Declaration of competing interest

The authors declare that they have no known competing financial interests or personal relationships that could have appeared to influence the work reported in this paper.
