# Supplementary material for: Carbon dot/polylactic acid nanofibrous membranes for solar-mediated oil absorption/separation: Performance, environmental sustainability, ecotoxicity and reusability
Source: Heliyon. 2024 Feb 16;10(4):e25417. doi: 10.1016/j.heliyon.2024.e25417 (PMC10900409; doi:10.1016/j.heliyon.2024.e25417)
Supplement: Multimedia component 1 [file mmc1.docx]

Supporting Information

**Carbon dot / polylactic acid nanofibrous membranes for solar-mediated oil absorption/separation: performance, environmental sustainability, ecotoxicity and reusability**

*Monica Torsello^a^, Shani Ben-Zichri^b^, Lucia Pesenti^a^, Sisira M. Kunnath^b^, Chiara Samorì^a^, Andrea Pasteris^c^, Greta Bacchelli^d^, Noa Prishkolnik^b^, Uri Ben-Nun^b^, Serena Righi^d,g^, Maria L. Focarete^a,e,f^, Sofiya Kolusheva^b^, Raz Jelinek^b^, Chiara Gualandi^a,,e,f,g*^, Paola Galletti^a^*

^a^ Department of Chemistry “Giacomo Ciamician”, University of Bologna, Via Selmi, 2, 40126 Bologna, Italy.

^b^ Department of Chemistry, Ben Gurion University of the Negev, Beer Sheva 84105, Israel.

^c^ Department of Biological, Geological and Environmental Sciences, University of Bologna, via Sant’Alberto 163, 48123, Ravenna, Italy.

^d^ Interdepartmental Centre for Research in Environmental Sciences (CIRSA), University of Bologna, Via S. Alberto, 163, 48123 Ravenna, Italy.

^e^ INSTM UdR of Bologna, University of Bologna, Via Selmi, 2, 40126 Bologna, Italy.

^f^ Health Sciences and Technologies – Interdepartmental Center for Industrial Research (HST-ICIR), Alma Mater Studiorum - Università di Bologna, 40064 Ozzano dell’Emilia, Bologna, Italy.

^g^ Interdepartmental Center for Industrial Research on Advanced Applications in Mechanical Engineering and Materials Technology, CIRI-MAM, University of Bologna, Viale Risorgimento, 2, 40136 Bologna, Italy.

^h^ Department of Physics and Astronomy “Augusto Righi”, University of Bologna, Viale Carlo Berti Pichat, 6/2, 40126 Bologna, Italy.

**Table S1**. Inventory data of CDs synthesis.

| **INPUTS** | **Values** |
| --- | --- |
| Citric acid [g] | 1.0 |
| Octadecylamine [g] | 1.0 |
| Urea [g] | 1.0 |
| Dimethylsulfoxide [mL] | 10 |
| DDW [L] | 10 |
| **PROCESS PARAMETERS** |  |
| Oven Temperature [°C] | 160 |
| Oven Power [W] | 1000 |
| Thermal decomposition duration [h] | 4 |
| Centrifugation rate [RPM] | 10000 |
| Centrifugation duration [h] | 0.17 (10 min) |
| Centrifugation electricity consumption [W] | 850 |
| DDW Water purifier electricity consumption [W] | 80 |
| Dialysis duration [h] | 48 |
| Magnetic stirrer electricity consumption [W] | 825 |
| Magnetic stirrer duration [h] | 48 |
| **OUTPUTS** |  |
| CDs [g] | 1.0 |
| Wastewater [L] | 10 |
| Waste solution [g] (solid precursors + solvent) | 12.0 |

**Table S2**. Inventory data of membrane preparation by electrospinning.

| **INPUTS** | **Value** |
| --- | --- |
| Poly-L-lactic acid [g/mL] | 0.15 |
| CDs [g/mL] | 0 (PLA)  7.9 (PLA-5CDs)  37.5 (PLA-20CDs)  100 (PLA-40CDs) |
| Dichloromethane [g/mL] | 0.9275 |
| N,N-dimethylformamide [g/mL] | 0.2832 |
| **OUTPUTS** |  |
| composite PLLA blank [mg] | 416.14 (3.4 mL) |
| composite PLLA+5wt%CDs [mg] | 271.32 (2.6 mL) |
| composite PLLA+20wt%CDs [mg] | 331.62 (3 mL) |
| composite PLLA+40wt%CDs [mg] | 336.16 (3 mL) |


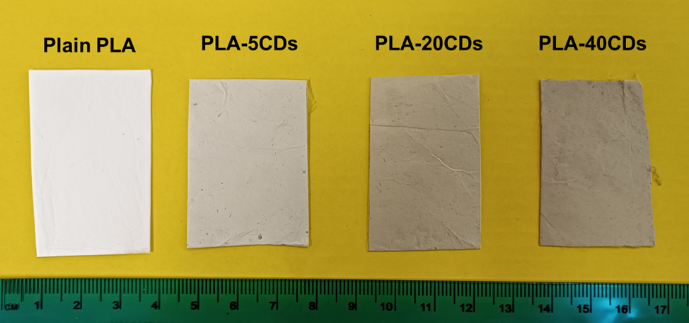


**Figure S1**. Pictures of electrospun membranes.


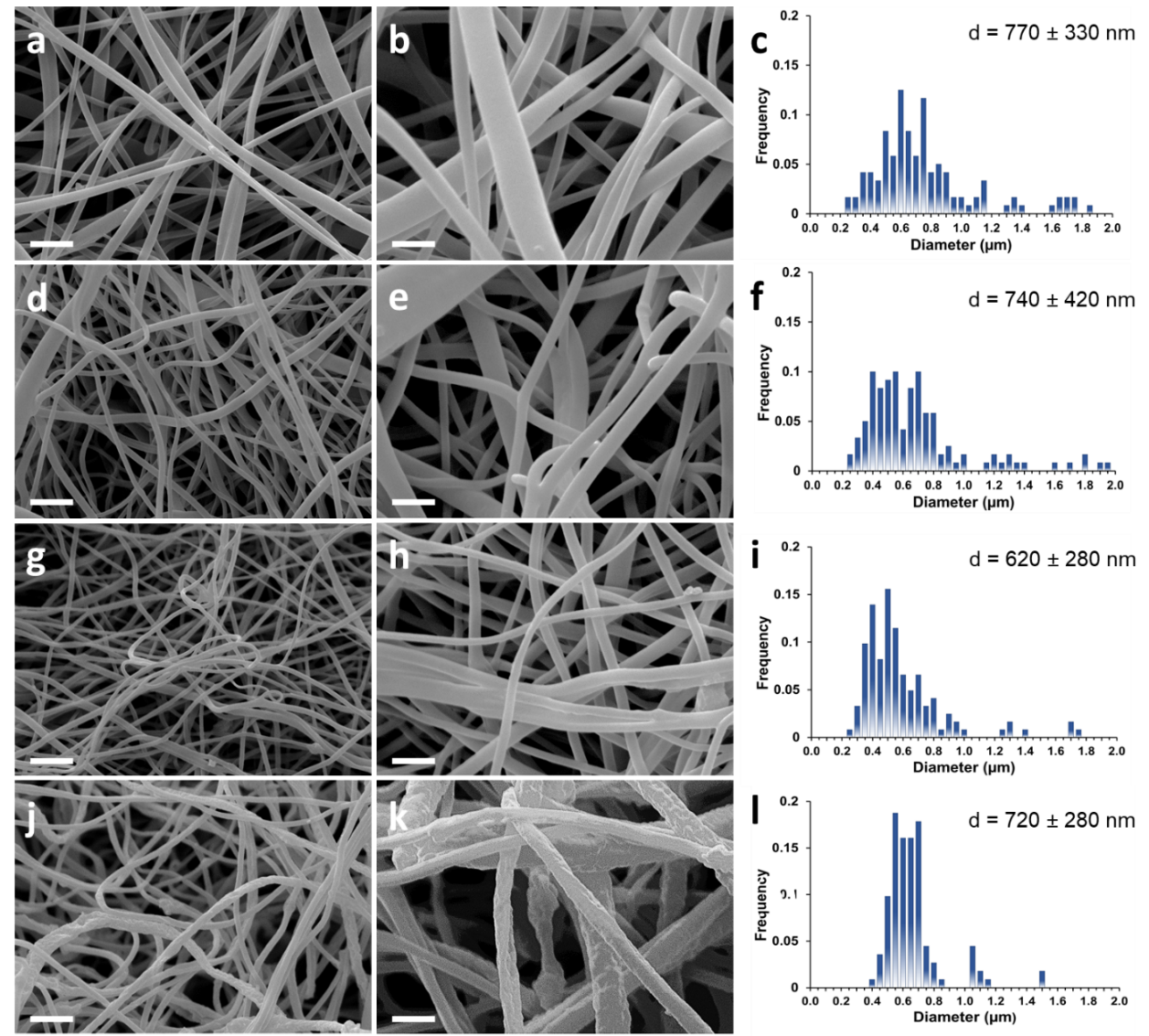


**Figure S2**. Representative SEM images of electrospun membranes: plain PLA (a, b); PLA-5CDs (d, e); PLA-20CDs (g, h); PLA-40CDs (j, k). Scale bars = 5 µm (a, d, g, j); 2 µm (b, e, h, k). Fiber diameter distribution of PLA (c), PLA-5CDs (f), PLA-20%CDs (i) and PLA-40%CDs (l).


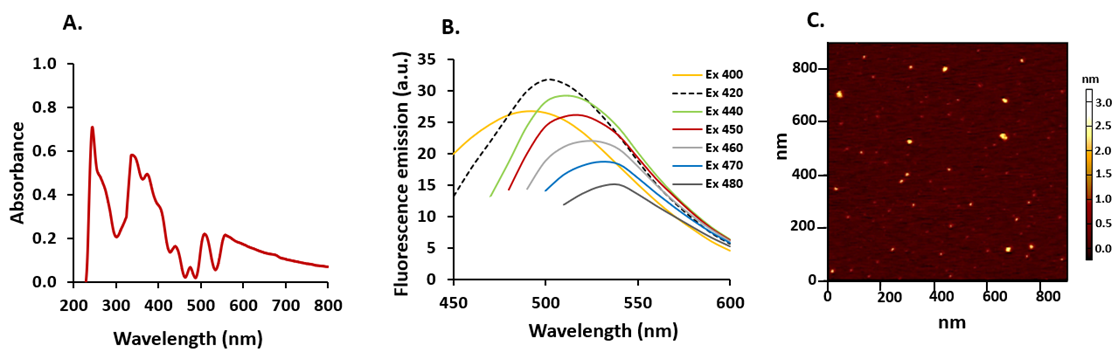


**Figure S3**. A) UV-Vis absorption spectrum of CDs. B) Excitation-dependent emission spectra of 100 µg mL^-1^ CDs in chloroform. C) Representative AFM image of 1 µg mL^-1^ CDs in chloroform deposited on a SiO_2_ substrate.


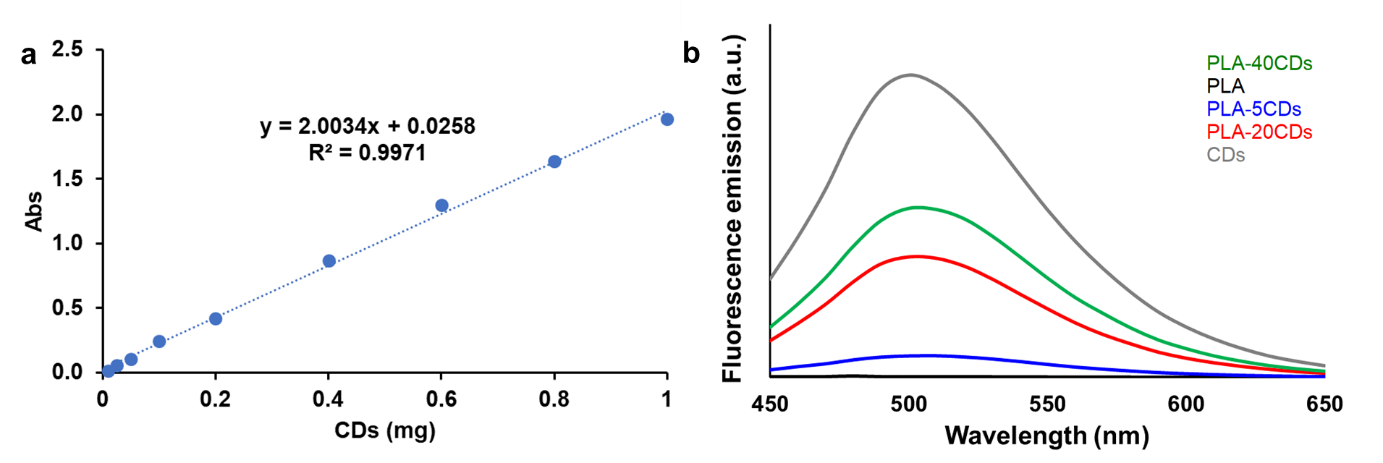


**Figure S4**. a) Calibration curve for the determination of CDs loading in the fibers by means of UV-VIS spectroscopy. b) Emission spectra of 0.33 mg membranes dissolved in 1 ml of DCM:DMF (70:30) v/v solvent mixture Ex wavelength was 420 nm, Em range was 450-650 nm.


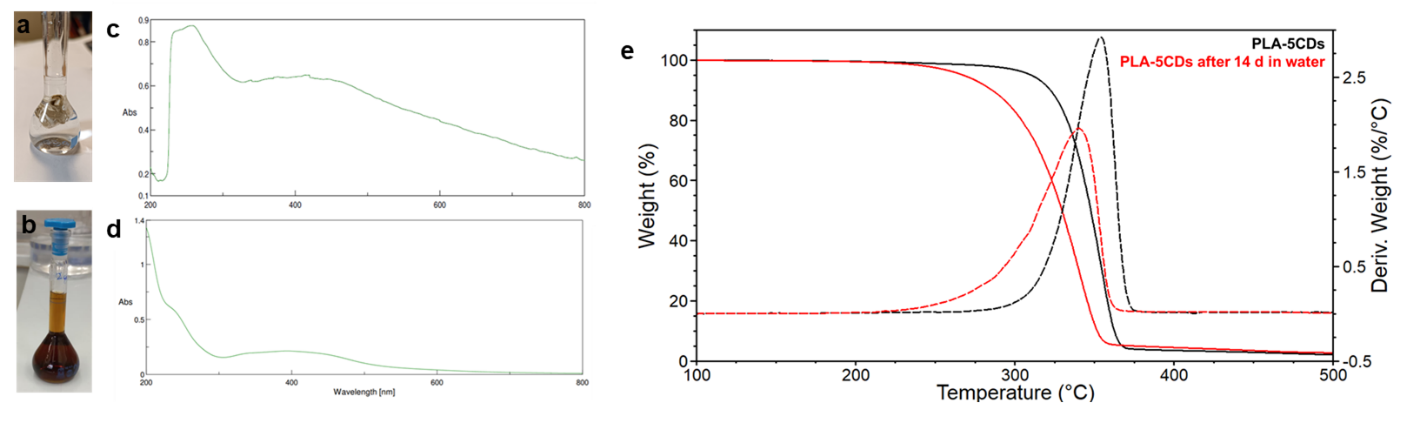


**Figure S5**. a) PLA-5CDs electrospun membrane immersed in water, b) aqueous solution after 24 h of contact with the PLA-5CDs, and its UV absorbance spectrum in c) dichloromethane and d) water. e) TGA (solid line) and corresponding derivative (dotted line) of PLA-5CDs before (black) and after (red) 14 days of water immersion.
